# Impact of Elevated Blood Pressure on Cardiovascular and Cerebrovascular Outcomes in Older Adults: A Propensity-Matched Analysis

**DOI:** 10.3390/jcm15145380

**Published:** 2026-07-09

**Authors:** Jigar Patel, Ronaldo Pichardo-Gonzalez, Samantha Camp, Quincy K. Tran, Adil Ather, Leah Steckler, Dominic S. Raj, Ali Pourmand

**Affiliations:** 1 Department of Internal Medicine, George Washington University School of Medicine and Health Sciences, Washington, DC 20037, USA; jigargj5@gmail.com (J.P.); ronaldopichardo@gmail.com (R.P.-G.); 2 Department of Emergency Medicine, University of Maryland School of Medicine, Baltimore, MD 21201, USA; samantha.camp@som.umaryland.edu (S.C.); qtran@som.umaryland.edu (Q.K.T.); 3 Program in Trauma, The R Adams Cowley Shock Trauma Center, University of Maryland School of Medicine, Baltimore, MD 21201, USA; 4 Department of Emergency Medicine, George Washington University School of Medicine and Health Sciences, 2120 L St., Washington, DC 20037, USA; adilather@gwmail.gwu.edu (A.A.); leah_steckler@gwu.edu (L.S.); 5 Division of Renal Diseases and Hypertension, George Washington University School of Medicine and Health Sciences, Washington, DC 20037, USA; draj@mfa.gwu.edu

**Keywords:** hypertension, older adults, systolic blood pressure, emergency department, mortality

## Abstract

**Introduction:** Hypertension (HTN) management is essential to improving cardiovascular and cerebrovascular morbidity and mortality in the older adult, yet optimal blood pressure (BP) targets remain uncertain. We aim to compare the incidence of major adverse cardiovascular events (MACE) among older adults presenting to the emergency department (ED) with systolic blood pressure (SBP) 140–159 mmHg and diastolic blood pressure (DBP) 90–99 mmHg versus those with SBP ≥ 160 mmHg and DBP ≥ 100 mmHg. **Methods:** Retrospective analysis was conducted using the Global Collaborative Network including adults aged ≥ 65 years presenting to the ED with essential HTN without end-stage renal disease. Cohort 1 (SBP ≥ 160 mmHg and DBP ≥ 100 mmHg) included older adults with more severely elevated BP, while Cohort 2 (SBP 140–159 mmHg and DBP 90–99 mmHg) included those with moderately elevated BP. The primary outcome was all-cause mortality over 5 years. Secondary outcomes included AKI, CHF, AMI, ischemic stroke, and hemorrhagic stroke. Propensity score matching was used to balance baseline characteristics. **Results:** After propensity score matching, 191,829 patients remained in each cohort. The mean age was 72.6 (±5.9) years; 51.8% were female, 10.5% had diabetes mellitus, and 5.65% were obese. Cohort 2 (SBP 140–159 mmHg) had lower risks across all primary and secondary outcomes compared with Cohort 1 (SBP ≥ 160 mmHg). The largest risk difference (RD) was observed for AKI (RD = 1.28%; 95% CI, 1.09–1.46%; *p* < 0.0001), followed by ischemic stroke (RD = 1.25%; 95% CI, 1.11–1.39%; *p* < 0.0001), CHF (RD = 1.11%; 95% CI, 0.93–1.29%; *p* < 0.0001), AMI (RD = 0.70%; 95% CI, 0.58–0.83%; *p* < 0.0001), all-cause mortality (RD = 0.33%; 95% CI, 0.15–0.51%; *p* < 0.0001), and hemorrhagic stroke (RD = 0.29%; 95% CI, 0.22–0.36%; *p* < 0.0001). All outcomes demonstrated consistently higher absolute risk in Cohort 1 compared with Cohort 2. **Conclusion:** Cohort 2 (SBP 140–159 mmHg) was associated with a statistically significant but small decrease in cardiovascular, cerebrovascular, and mortality outcomes, with all absolute RDs uniformly small (<1%) and minimal survival benefit over time. These small gains must be weighed against overtreatment risks, such as medication costs, hypotension, and falls, underscoring the need for individualized, risk-based HTN management in older adults.

## 1. Introduction

Hypertension (HTN) management is essential for reducing cardiovascular and cerebrovascular morbidity and mortality in older adults, yet optimal blood pressure (BP) targets remain uncertain. The prevalence of HTN increases dramatically with age, affecting approximately 70% of adults aged 65 years and older in the United States [1,2]. This age-related increase in BP, coupled with a growing population of older adults, presents significant challenges for clinical management and public health policy alike.

The financial burden of hypertension in the United States is substantial, with national healthcare expenditures attributable to HTN estimated at $131 billion annually, a figure disproportionately driven by older adults who carry the highest comorbidity burden [3] U.S. Census Bureau projections indicate that by 2034, adults aged 65 and older will outnumber those under 18 for the first time in the nation’s history, suggesting that the population-level burden of HTN-related cardiovascular disease will continue to grow [4]. Isolated systolic hypertension, defined as SBP ≥ 140 mmHg with DBP below 90 mmHg, accounts for the predominant form of hypertension in adults over age 60 and reflects progressive loss of aortic compliance and large artery stiffening that accompanies aging [5]. This hemodynamic profile distinguishes older hypertensive adults from younger patients and has direct implications for how BP targets and treatment risks are weighed in this population.

Aging is accompanied by physiological changes that directly affect how hypertension should be managed in older adults. Baroreceptor sensitivity declines with age, reducing the capacity to buffer acute BP fluctuations and predisposing patients to both hypertensive surges and orthostatic hypotension [6]. In patients with long-standing hypertension, the lower limit of cerebral autoregulation shifts upward, such that BP reductions that appear pharmacologically appropriate may fall below the threshold at which the brain can independently maintain adequate perfusion, contributing to the J-curve relationship between BP and adverse outcomes observed in several large trials [7]. Additionally, polypharmacy is highly prevalent in this population, and antihypertensive agents are frequently co-prescribed with medications for heart failure, chronic kidney disease, and Parkinson’s disease, increasing the risk of drug–drug interactions, electrolyte disturbances, and renal injury [8]. These factors collectively support an individualized rather than uniform approach to BP targets in older adults.

Contemporary guidelines increasingly recognize that age alone is insufficient to determine an appropriate BP target. The 2025 AHA/ACC guideline recommends a target of <130/80 mmHg for most adults (including those aged 65 and older) while allowing a more permissive goal of ≤140/90 mmHg in patients aged 80 or older and in those with frailty, orthostatic hypotension, or significant multimorbidity [9]. The SPRINT and STEP trials provided important evidence supporting intensive BP control in older adults; however, both studies excluded patients with diabetes, prior stroke, advanced CKD, and significant frailty [10,11], which are characteristics that are common among older adults presenting to the emergency department (ED). Notably, SPRINT employed automated unattended oscillometric BP measurements, a method that yields readings approximately 5 to 10 mmHg lower than conventional office measurements [10], further limiting the direct applicability of trial-derived thresholds to the acute care setting. These limitations leave considerable uncertainty regarding optimal BP management for the heterogeneous older adult population encountered in the ED.

Elevated BP exerts its pathological effects through multiple mechanisms that directly contribute to cardiovascular morbidity and mortality. Chronic HTN accelerates atherosclerosis by promoting endothelial dysfunction, increasing arterial stiffness, and enhancing oxidative stress within vessel walls [12,13]. This process leads to the development of coronary artery disease, carotid stenosis, and peripheral arterial disease. Additionally, sustained pressure overload causes left ventricular hypertrophy and diastolic dysfunction, predisposing patients to heart failure with preserved ejection fraction, a condition particularly prevalent in older populations [14,15].

The cerebrovascular consequences of hypertension are equally profound. Elevated BP damages small penetrating arteries in the brain through lipohyalinosis and microatheroma formation, leading to lacunar strokes and white matter hyperintensities [16]. Large-vessel cerebrovascular disease develops through similar atherosclerotic mechanisms affecting carotid and vertebrobasilar circulation [17,18]. Furthermore, hypertension weakens cerebral blood vessel walls, increasing the risk of hemorrhagic stroke through the development of microaneurysms and arteriovenous malformations [19,20].

Despite these well-known pathophysiological mechanisms, optimal BP targets for this patient population remain uncertain, creating challenges for clinical management, especially in the acute care setting. The ED provides a unique window into HTN management challenges in older adults. Older adults frequently present to the ED with elevated BP, either as a primary complaint or as an incidental finding during evaluation for other conditions [21,22]. The acute care environment often reveals patients with poorly controlled hypertension or those experiencing hypertensive crises, making it an important venue for understanding real-world cardiovascular risk patterns. The prognostic significance of BP measured at ED presentation for long-term cardiovascular outcomes remains poorly understood.

Contemporary hypertension management guidelines emphasize the importance of individualized risk assessment and shared decision-making, particularly in older adults [9]. However, the evidence base supporting specific BP targets in this patient population remains limited, creating uncertainty for clinicians and patients alike. Large-scale, real-world studies examining long-term outcomes across different hypertension stages can provide valuable insights to inform clinical practice and guideline development.

We aimed to compare the five-year risk of adverse cardiovascular and cerebrovascular outcomes among older adults presenting to the ED with SBP ≥ 160 mmHg and DBP ≥ 100 mmHg (Cohort 1) versus those presenting with SBP 140–159 mmHg and DBP 90–99 mmHg (Cohort 2).

## 2. Methods

### 2.1. Study Design and Data Source

This is a retrospective, propensity-matched cohort study using the TriNetX research network (https://trinetx.com/). TriNetX is a large database that includes data from 144 large healthcare organizations (HCOs) with more than 130 million patients from the Americas, Europe, and Asia. This study includes data from 1 January 2016 to 15 April 2026. The database comprises de-identified, de-aggregated health records and includes data on demographics, admissions, medications, procedures, diagnoses, and laboratory values. These data points are entered into TriNetX with standard classifications including International Classification of Diseases (ICD) for diagnoses, ICD and Current Procedural Terminology (CPT) for procedures, RxNorm for medications, and Logical Observation Identifiers Names and Codes (LOINCs) for laboratory test results. This study followed the Strengthening the Reporting of Observational Studies in Epidemiology (STROBE) guidelines for cohort studies [23].

### 2.2. Participants

This study included all patients aged 65 years and older who presented to an ED with an existing diagnosis of essential (primary) hypertension (ICD10CM:I10). Cohorts were stratified according to BP measurements within predefined ranges recorded on the same day as the ED visit. Cohort 1 comprised patients with a SBP ≥ 160 mmHg and a DBP ≥ 100 mmHg (stage 2 or greater hypertension by AHA classification). Cohort 2 comprised patients with a SBP 140–159 mmHg and a DBP 90–99 mmHg (stage 1 hypertension by AHA classification). This classification aligns with the 2025 AHA/ACC guideline staging framework and provides a clinically meaningful distinction between moderate and more severely elevated BP at the time of ED presentation. Patients were excluded from both cohorts if they had a diagnosis of end-stage renal disease (ESRD) (ICD10CM:N18.6) present at the time of ED presentation. Patients were excluded from analysis of specific outcomes if they had any instance of that outcome prior to the index event.

### 2.3. Outcome Measurements

The primary outcome was all-cause mortality over 5 years. Secondary outcomes included risk of cardiovascular and cerebrovascular events. Cardiovascular events were defined as congestive heart failure (CHF) (ICD10CM:I50) and acute myocardial infarction (including STEMI and NSTEMI) (ICD10CM:I21, ICD10CM:I21.3, ICD10CM:I21.4). Cerebrovascular events included ischemic stroke (ICD10CM:I63, ICD10CM:I63.50) and hemorrhagic stroke (ICD10CM:I62.9, ICD10CM:I60, ICD10CM:I60.7, ICD10CM:I61). Acute kidney injury (AKI) (ICD10CM:N17) was additionally included as a secondary outcome given its established association with hypertensive end-organ damage.

### 2.4. Statistical Analysis

Descriptive statistics were presented as mean ± standard deviation (SD), or frequency (%). Propensity score matching was performed on all listed characteristics. Cohorts were matched using the TriNetX propensity-matching tool to match Cohort 1 (SBP ≥ 160 mmHg) and Cohort 2 (SBP 140–159 mmHg). Demographic variables, including age, sex, race, and ethnicity, as well as laboratory values (Blood Urea Nitrogen, Creatinine, Cholesterol [LDL, HDL], Triglyceride, HemoglobinA1c) and pre-existing comorbidities, including diabetes mellitus, obesity, and dyslipidemia, were matched between cohorts. The TriNetX propensity matching algorithm uses “greedy nearest neighbor matching” with a caliper of 0.1 to match patients as 1:1 pairs. After determining the matched cohorts, risk differences (RDs) were calculated. Comparisons of outcomes between propensity score-matched groups were expressed as risk difference, the 95% confidence interval (95% CI), and the associated *p*-values. All tests with *p*-value < 0.05 were considered statistically significant. Statistical analysis was completed using built-in TriNetX software. Matching variables were selected based on their established association with both hypertension severity and adverse cardiovascular outcomes; diabetes mellitus, obesity, and dyslipidemia were specifically included given their well-documented roles as independent cardiovascular and cerebrovascular risk factors that could confound the relationship between blood pressure levels and outcomes [14].

## 3. Ethics

The study was exempted from formal consent by the IRB of the University of Maryland Baltimore as it was considered non-human subject research (HP-00117259).

## 4. Results

### 4.1. Patient Demographics

A total of 484,701 patients met the inclusion and exclusion criteria for this study. Prior to matching, 192,360 patients met the criteria for Cohort 1 (SBP ≥ 160 mmHg) and 291,341 patients met the criteria for Cohort 2 (SBP 140–159 mmHg). Post-match baseline characteristics are presented in Table 1. After propensity score matching, 191,829 matched pairs were retained for outcome analysis.

The average age (±SD) at index for individuals in both matched cohorts was 72.6 (±5.9) years. Approximately 51.8% of Cohort 1 and 51.7% of Cohort 2 were female, 61.6% were White, and 15.7% were Black. The prevalence of diabetes mellitus (10.5% vs. 10.3%), obesity (5.6% vs. 5.5%), and dyslipidemia (21.9% vs. 21.8%) was similar between cohorts after matching, confirming adequate balance.

### 4.2. Primary Outcome

The primary outcome was all-cause mortality. Over five years, mortality occurred in 17,464 patients in Cohort 1 (SBP ≥ 160 mmHg) and 16,873 patients in Cohort 2 (SBP 140–159 mmHg), corresponding to a small but statistically significant absolute risk difference of 0.33% (95% CI, 0.15–0.51%; *p* < 0.0001).

### 4.3. Secondary Outcomes

All secondary outcomes occurred at higher rates in Cohort 1 (SBP ≥ 160 mmHg) than in Cohort 2 (SBP 140–159 mmHg), with statistically significant differences observed across all cardiovascular, cerebrovascular, and renal endpoints. The incidence of AKI was 9.5% in Cohort 1 and 8.3% in Cohort 2, with a risk difference of 1.28% (95% CI, 1.09–1.46%; *p* < 0.0001). For CHF, the event rate was 8.8% vs. 7.7%, yielding a risk difference of 1.11% (95% CI, 0.93–1.29%; *p* < 0.0001). MI occurred in 4.1% of patients in Cohort 1 compared to 3.4% in Cohort 2, with a risk difference of 0.70% (95% CI, 0.58–0.83%; *p* < 0.0001). Ischemic stroke occurred in 5.4% of Cohort 1 versus 4.2% of Cohort 2, corresponding to a risk difference of 1.25% (95% CI, 1.11–1.39%; *p* < 0.0001). Hemorrhagic stroke was less frequent overall, with event rates of 1.4% and 1.1%, respectively, translating to a risk difference of 0.29% (95% CI, 0.22–0.36%; *p* < 0.0001). Among all secondary outcomes, AKI and ischemic stroke demonstrated the largest absolute risk differences at 1.28% and 1.25%, respectively. A complete summary of all primary and secondary outcome event rates, risk differences, and confidence intervals is presented in Table 2.

### 4.4. Subgroup Analysis

Table 3 presents the age-stratified subgroup analysis after propensity score matching. The association between Cohort 1(BP > 160 mmHg) and adverse outcomes was higher among patients aged 65–74 years and progressively attenuated with advancing age. Among adults aged 65–74 years, Cohort 1 had significantly higher risks of acute kidney injury (RD 1.4%, 95% CI 1.2–1.6), congestive heart failure (RD 1.2%, 95% CI 1.0–1.4), acute myocardial infarction (RD 0.7%, 95% CI 0.6–0.9), hemorrhagic stroke (RD 0.3%, 95% CI 0.2–0.4), ischemic stroke (RD 1.3%, 95% CI 1.1–1.4), and all-cause mortality (RD 0.5%, 95% CI 0.3–0.7) compared with Cohort 2 (all *p* < 0.0001). Similar but smaller differences were observed among patients aged 75–84 years. In contrast, among adults aged ≥ 85 years, no statistically significant differences were observed between cohorts for any of the evaluated outcomes, with risk differences approaching zero across all endpoints (Table 4).

## 5. Discussion

In this retrospective cohort study of older adults presenting to the ED with hypertension, we found that those in Cohort 1 (SBP ≥ 160 mmHg and DBP ≥ 100 mmHg) had greater risks of adverse cardiovascular and renal outcomes, including ischemic stroke, congestive heart failure, acute kidney injury, myocardial infarction, and hemorrhagic stroke, compared with individuals in Cohort 2 (SBP 140–159 mmHg and DBP 90–99 mmHg). These associations were modest yet remained statistically significant after propensity score matching.

In a recent ED-based cohort study of patients with severe hypertension (SBP ≥ 180 mmHg) without acute end-organ damage, Chaudhry et al. found that discharge SBP ≤ 160 mmHg did not confer a reduction in 30-day or one-year major adverse cardiovascular events (MACE) [24]. These results suggest that elevated BP readings in the ED should be interpreted within the broader clinical context, as they may represent acute, reversible responses, for example, pain or anxiety, rather than chronic hypertension. This is consistent with our finding of minimal risk differences between Cohort 1 and Cohort 2 across a five-year follow-up horizon.

Our findings align with prior randomized controlled trials and observational studies examining the relationship between BP and cardiovascular outcomes in older adults. The SPRINT trial demonstrated that targeting a systolic BP < 120 mmHg significantly reduced major cardiovascular events compared with standard control (<140 mmHg) among adults aged ≥ 50 years with elevated cardiovascular risk but without diabetes or prior stroke [10]. Similarly, the STEP trial found reduced adverse events with SBP 110–130 mmHg compared with SBP 130–150 mmHg in healthy adults aged 60–80 without major comorbidities such as heart failure or advanced kidney disease [11]. Together, these trials support the cardiovascular benefits of intensive BP control in healthier older adults. In contrast, our study population included a more heterogeneous mix of patients with diabetes, CKD, and multiple comorbidities, producing more modest outcome differences that reflect the complexity of routine clinical practice.

Recent clinical policy from the American College of Emergency Physicians (ACEP) emphasizes that for adults presenting to the ED with asymptomatic elevated BP, routine acute pharmacologic lowering is not recommended [25]. Our results support this policy, as we observed only modest absolute differences in long-term outcomes between Cohort 2 (stage 1 HTN, SBP 140–159 mmHg) and Cohort 1 (stage 2 or greater HTN, SBP ≥ 160 mmHg). In fact, overly aggressive pharmacologic reduction in the ED may be harmful in older adults and has been associated with increased risk of adverse events [26]. Frailty, which is present in up to 40% of older adults presenting to the emergency department, is an independent predictor of adverse outcomes and may substantially modify the cardiovascular risk associated with elevated blood pressure [27].

The observation that AKI demonstrated the largest absolute risk difference among all outcomes (RD = 1.28%) may be explained by the cumulative renal effects of sustained hypertension. Chronic HTN promotes glomerulosclerosis through efferent arteriolar vasoconstriction and sustained intraglomerular hypertension, progressively reducing nephron mass and renal autoregulatory reserve [28]. Patients in Cohort 1, with more severely elevated BP at ED presentation, likely carry a greater burden of underlying hypertensive nephrosclerosis, rendering them more susceptible to acute kidney injury when superimposed stressors such as infection, volume depletion, or decompensated heart failure are present. Additionally, reduced eGFR from hypertensive renal injury further activates the renin–angiotensin–aldosterone system (RAAS), worsening BP control and perpetuating a cycle of cardiorenal deterioration [28]. The renal risk difference in our cohort exceeded that of ischemic stroke, suggesting that AKI may serve as a particularly sensitive marker of cumulative hypertensive end-organ damage in older adults presenting to the ED.

Several factors may explain the uniformly small absolute risk differences observed between cohorts. First, a single BP measurement obtained in the ED may not accurately reflect chronic BP burden, as acute illness, pain, and anxiety can all transiently elevate BP independent of underlying hypertension severity. Clinic-based and ambulatory BP measurements demonstrate poor correlation, particularly in older adults with significant BP variability [29], and this misclassification may have attenuated the observed between-group differences. Second, pharmacological treatment modifications following the ED visit likely reduce differences over time, as patients with documented hypertension frequently undergo medication initiation or dose adjustment after discharge. Third, in older patients with multiple comorbidities, non-cardiovascular causes of death may predominate, reducing the apparent contribution of elevated BP to long-term mortality. These findings are also consistent with growing evidence that visit-to-visit BP variability, rather than mean BP alone, is an independent predictor of adverse cardiovascular outcomes [29].

Although the differences between cohorts reached statistical significance, the absolute risk differences were uniformly small for hemorrhagic stroke, AKI, and all-cause mortality. These findings highlight the important distinction between statistical and clinical significance and caution against interpreting the observed associations as justification for more aggressive acute blood pressure lowering in older adults presenting to the ED.

Sex- and race-based differences in hypertension-related outcomes represent an important dimension not fully captured by our aggregate analysis. Women comprised approximately 52% of both cohorts and exhibit a distinct hypertension phenotype, with steeper age-related increases in SBP beyond the sixth decade, higher rates of heart failure with preserved ejection fraction, and greater BP variability compared with men [30]. Prior trial data, including the ALLHAT study, suggest that women may respond differently to specific antihypertensive drug classes than men, with implications for individualized treatment selection [31]. Among racial groups, Black patients face a disproportionately high prevalence of early-onset severe hypertension, greater end-organ damage at comparable BP levels, and lower rates of BP control, attributed in part to genetic polymorphisms affecting the renin–angiotensin–aldosterone system and to the downstream effects of socioeconomic disadvantage on cardiovascular health [31]. Future analyses should include dedicated subgroup evaluations by sex, race, and socioeconomic status to better characterize differential risk and refine treatment thresholds across these populations.

These findings have direct implications for HTN management in the acute care setting. While the consistently higher event rates observed in Cohort 1 support the importance of recognizing and documenting severely elevated BP during ED visits, the small absolute risk differences observed over five years suggest that aggressive acute pharmacologic lowering is unlikely to substantially alter long-term cardiovascular outcomes and may carry harm in older adults, as previously described [26]. Rather, the ED visit may represent an important opportunity for medication reconciliation, risk communication, and facilitation of timely outpatient follow-up to establish or optimize a strong antihypertensive regimen. Pharmacist-led transitional care programs linking the ED to outpatient hypertension clinics have demonstrated feasibility and clinically meaningful BP reductions in high-risk populations lacking consistent primary care access [32]. These programs represent a promising model for addressing the gap between acute BP identification and sustained long-term management.

Older adults with dementia tend to visit the ED more frequently than others in their age cohort without cognitive impairment; however, documentation of known dementia is often not captured [33,34]. Furthermore, when assessing for frailty, there are myriad scoring systems and overall limited clinical utility of the current documentation means [35]. In conjunction with the limitations of the data set regarding hypotension and fall history, to further understand outcomes in older adults who present to the ED with elevated blood pressure, more reliable and standardized measures of cognition and frailty need to be in place. Recent CMS requirements surrounding age-friendly health systems may enhance capture of this information in future research.

The relationship between BP and cardiovascular outcomes is complex and continues to evolve. While the benefits of BP reduction are well-established in younger populations and in carefully selected older adults, the optimal BP targets for the heterogeneous older adult encountered in real-world acute care settings remain uncertain, and our findings contribute to this ongoing discussion.

## 6. Future Directions

Future research should focus on prospective studies evaluating optimal blood pressure targets in older adults presenting to the emergency department, particularly in the context of acute illness, frailty, and multimorbidity. While our findings demonstrated statistically significant differences in cardiovascular and cerebrovascular outcomes between Cohort 1 (SBP ≥ 160 mmHg) and Cohort 2 (SBP 140–159 mmHg), the absolute risk reductions were uniformly small. These findings suggest that standardized aggressive BP lowering strategies may not provide substantial long-term benefit for many older adults and reinforce the need for individualized treatment approaches. Prospective investigations incorporating serial blood pressure measurements, frailty indices, functional status, medication adherence, and post-discharge follow-up are needed to better distinguish transient ED-associated BP elevations from chronic uncontrolled hypertension.

Additionally, future studies should evaluate patient-centered outcomes beyond traditional cardiovascular endpoints, including quality of life, falls, medication-related adverse events, cognitive decline, and healthcare utilization. Subgroup analyses examining differences by age strata, sex, race and ethnicity, chronic kidney disease, and baseline cardiovascular risk may further refine risk stratification and therapeutic thresholds in older adults. Given the increasing emphasis on precision medicine and shared decision-making, future research should also explore risk-based ED management pathways and determine whether selective outpatient follow-up strategies may provide safer and more cost-effective alternatives to routine acute pharmacologic intervention for asymptomatic elevated blood pressure in the emergency setting.

## 7. Limitations

This study has several limitations that should be considered. First, the study is retrospective in nature and relies on EMR data from the TriNetX network, which introduces inherent risks of residual confounding and selection bias despite propensity score matching. We cannot exclude the possibility that unmeasured variables, such as socioeconomic status and functional status, influenced the observed findings. Second, BP measurements were obtained during ED visits, which may reflect transient hemodynamic responses to acute illness, pain, or anxiety rather than chronic hypertension severity. This limits the ability to determine whether the observed differences in outcomes between Cohort 1 and Cohort 2 are attributable to true differences in baseline BP burden. Third, our dataset lacked granular clinical detail on antihypertensive medication use, dose adjustments, and longitudinal BP control following the index ED encounter. As such, we were unable to assess how post-ED BP management may have influenced long-term outcomes. The observed associations may reflect confounding by unmeasured variables that we were unable to match for, including functional status, cognitive impairment, fall history, and orthostatic hypotension, all of which are highly prevalent in older adults and independently influence both blood pressure and clinical outcomes. Finally, we were unable to assess medication adherence or post-discharge antihypertensive compliance, which may have influenced the observed outcomes. Prospective studies incorporating longitudinal medication and adherence data are needed to clarify this relationship. These limitations underscore the need for prospective studies incorporating serial BP measurements, medication data, comorbidity burden, and full clinical context to refine risk stratification in older adults with elevated BP in acute care settings.

## 8. Conclusions

Study subjects in Cohort 2 (SBP 140–159 mmHg) demonstrated a statistically significant but small decrease in cardiovascular, cerebrovascular, and mortality outcomes compared with Cohort 1 (SBP ≥ 160 mmHg), with all absolute risk differences uniformly small (<1%) and minimal survival benefit over time. These modest benefits of reduced BP must be balanced against the potential harms of overtreatment, including medication costs, polypharmacy, hypotension, and falls, particularly in older adults. In the acute care setting, where elevated BP may be transient and follow-up uncertain, our findings support a more individualized, risk-based approach to management rather than uniform or standardized aggressive blood pressure-lowering treatment.

## Figures and Tables

**Table 1:** Baseline demographics after propensity score matching.

Variable	Cohort 1 (>160 mmHg) N = 191,829	Cohort 2 (140–159 mmHg) N = 191,829	*p*-Value	Std Diff
**Age**				
Mean ± SD	72.6 ± 5.9	72.6 ± 5.9	0.619	0.002
**Sex, n (%)**				
Male	92,460 (48.2%)	92,640 (48.3%)	0.561	0.002
Female	99,347 (51.8%)	99,165 (51.7%)	0.557	0.002
**Race, n (%)**				
White	118,164 (61.6%)	118,251 (61.6%)	0.773	0.001
Black or African American	30,148 (15.7%)	30,163 (15.7%)	0.947	<0.001
Asian	8227 (4.3%)	8182 (4.3%)	0.720	0.001
Native Hawaiian or Other Pacific Islander	1476 (0.8%)	1435 (0.7%)	0.446	0.002
American Indian or Alaska Native	596 (0.3%)	540 (0.3%)	0.096	0.005
Hispanic or Latino	6288 (3.3%)	6122 (3.2%)	0.130	0.005
Not Hispanic or Latino	142,989 (74.5%)	143,391 (74.7%)	0.136	0.005
**Co-Morbidities, n (%)**				
Diabetes Mellitus	20,182 (10.5%)	19,740 (10.3%)	0.019	0.008
Overweight and Obesity	10,697 (5.6%)	10,570 (5.5%)	0.370	0.003
Disorders of Lipoprotein Metabolism	42,096 (21.9%)	41,787 (21.8%)	0.227	0.004
Acute Kidney Failure	3042 (1.6%)	2674 (1.4%)	<0.001	0.016

Note: Cohort 2 includes patients presenting to the emergency department with systolic blood pressure (SBP) 140–159 mmHg and diastolic blood pressure (DBP) 90–99 mmHg. Cohort 1 includes patients with SBP ≥ 160 mmHg and DBP ≥100 mmHg. The data are presented as mean ± standard deviation (SD) or numbers (percentages), as appropriate.

**Table 2:** Patient outcomes after propensity score matching.

Outcome	Cohort 1 (>160 mmHg)	Cohort 2 (140–159 mmHg)	Risk Difference % (95% CI)	*p*-Value
AKI, n (%)	17,341 (9.5%)	15,101 (8.3%)	+1.3% (1.1, 1.5)	<0.001
CHF, n (%)	15,669 (8.8%)	13,733 (7.7%)	+1.1% (0.9, 1.3)	<0.001
Acute MI, n (%)	7611 (4.1%)	6333 (3.4%)	+0.7% (0.6, 0.8)	<0.001
Hemorrhagic Stroke, n (%)	2654 (1.4%)	2108 (1.1%)	+0.3% (0.2, 0.4)	<0.001
Ischemic Stroke, n (%)	9713 (5.4%)	7598 (4.2%)	+1.3% (1.1, 1.4)	<0.001
All-cause Mortality, n (%)	17,464 (9.2%)	16,873 (8.8%)	+0.3% (0.2, 0.5)	<0.001

Note: Cohort 1 includes patients with SBP ≥ 160 mmHg and DBP ≥ 100 mmHg. Cohort 2 includes patients presenting to the emergency department with systolic blood pressure (SBP) 140–159 mmHg and diastolic blood pressure (DBP) 90–99 mmHg. AKI = acute kidney injury; CHF = congestive heart failure; MI = myocardial infarction; CI = confidence interval.

**Table 3:** Subgroup analysis after propensity score matching.

Age Group	65–74 Years Old				75–84 Years Old				≥85 Years Old			
Variable	Cohort 1 (>160 mmHg) N = 120,366	Cohort 2 (140–159 mmHg) N = 120,366	*p*-Value	Std Diff	Cohort 1 (>160 mmHg) N = 54,135	Cohort 2 (140–159 mmHg) N = 54,135	*p*-Value	Std Diff	Cohort 1 (>160 mmHg) N = 5660	Cohort 2 (140–159 mmHg) N = 5660	*p*-Value	Std Diff
**Age**												
Mean ± SD	68.9 ± 2.8	68.9 ± 2.8	0.893	0.001	78.6 ± 2.7	78.6 ± 2.7	0.909	0.001	86.3 ± 1.3	86.3 ± 1.3	0.730	0.006
**Sex, n (%)**												
Male	62,393 (51.8%)	62,392 (51.8%)	0.997	<0.001	23,352 (43.1%)	23,475 (43.4%)	0.451	0.005	2036 (36.0%)	2044 (36.1%)	0.876	0.003
Female	57,959 (48.2%)	57,960 (48.2%)	0.997	<0.001	30,776 (56.9%)	30,654 (56.6%)	0.454	0.005	3624 (64.0%)	3615 (63.9%)	0.860	0.003
**Race, n (%)**												
White	70,768 (58.8%)	70,998 (59.0%)	0.341	0.004	34,459 (63.7%)	34,521 (63.8%)	0.695	0.002	3887 (68.7%)	3883 (68.6%)	0.935	0.002
Black or African American	20,474 (17.0%)	20,448 (17.0%)	0.888	0.001	6026 (11.1%)	5990 (11.1%)	0.728	0.002	488 (8.6%)	503 (8.9%)	0.618	0.009
Asian	5429 (4.5%)	5353 (4.4%)	0.454	0.003	2625 (4.8%)	2602 (4.8%)	0.744	0.002	258 (4.6%)	265 (4.7%)	0.754	0.006
Native Hawaiian or Other Pacific Islander	1085 (0.9%)	1028 (0.9%)	0.213	0.005	379 (0.7%)	371 (0.7%)	0.769	0.002	16 (0.3%)	11 (0.2%)	0.335	0.018
American Indian or Alaska Native	277 (0.2%)	267 (0.2%)	0.668	0.002	115 (0.2%)	103 (0.2%)	0.416	0.005	11 (0.2%)	10 (0.2%)	0.827	0.004
Hispanic or Latino	4431 (3.7%)	4270 (3.5%)	0.079	0.007	1501 (2.8%)	1439 (2.7%)	0.246	0.007	137 (2.4%)	129 (2.3%)	0.620	0.009
Not Hispanic or Latino	87,341 (72.6%)	87,637 (72.8%)	0.176	0.006	39,357 (72.7%)	39,496 (73.0%)	0.342	0.006	4162 (73.5%)	4204 (74.3%)	0.369	0.017
**Co-Morbidities, n (%)**												
Diabetes Mellitus	11,951 (9.9%)	11,722 (9.7%)	0.117	0.006	5989 (11.1%)	5851 (10.8%)	0.179	0.008	510 (9.0%)	512 (9.0%)	0.948	0.001
Overweight and Obesity	7346 (6.1%)	7289 (6.1%)	0.627	0.002	2511 (4.6%)	2437 (4.5%)	0.282	0.007	176 (3.1%)	161 (2.8%)	0.407	0.016
Disorders of Lipoprotein Metabolism	24,287 (20.2%)	24,180 (20.1%)	0.587	0.002	12,842 (23.7%)	12,741 (23.5%)	0.470	0.004	1340 (23.7%)	1337 (23.6%)	0.947	0.001
Acute Kidney Failure	1656 (1.4%)	1429 (1.2%)	<0.001	0.017	866 (1.6%)	779 (1.4%)	0.031	0.013	97 (1.7%)	85 (1.5%)	0.370	0.017

**Table 4:** Subgroup analysis outcomes after propensity score matching.

Age Group	65–74 Years Old				75–84 Years Old				≥85 Years Old			
Outcome	Cohort 1	Cohort 2	RD % (95% CI)	*p*-Value	Cohort 1	Cohort 2	RD % (95% CI)	*p*-Value	Cohort 1	Cohort 2	RD % (95% CI)	*p*-Value
AKI, n (%)	9704 (8.5%)	8085 (7.0%)	+1.5% (1.2, 1.7)	<0.0001	5526 (10.9%)	5070 (9.9%)	+0.9% (0.6, 1.3)	<0.0001	502 (9.5%)	497 (9.4%)	+0.2% (−1.0, 1.3)	0.7846
CHF, n (%)	8412 (7.4%)	6996 (6.1%)	+1.3% (1.1, 1.5)	<0.0001	5303 (10.8%)	4940 (10.1%)	+0.7% (0.3, 1.1)	0.0003	494 (10.0%)	514 (10.6%)	−0.6% (−1.8, 0.6)	0.3140
Acute MI, n (%)	4331 (3.7%)	3428 (2.9%)	+0.8% (0.6, 0.9)	<0.0001	2326 (4.5%)	2108 (4.1%)	+0.4% (0.2, 0.7)	0.0007	230 (4.3%)	209 (3.9%)	+0.4% (−0.3, 1.1)	0.2905
Hemorrhagic Stroke, n (%)	1517 (1.3%)	1106 (0.9%)	+0.3% (0.3, 0.4)	<0.0001	905 (1.7%)	762 (1.4%)	+0.3% (0.1, 0.4)	0.0003	76 (1.4%)	83 (1.5%)	−0.1% (−0.6, 0.3)	0.6191
Ischemic Stroke, n (%)	5518 (4.9%)	4086 (3.6%)	+1.3% (1.2, 1.5)	<0.0001	3136 (6.3%)	2613 (5.2%)	+1.2% (0.9, 1.4)	<0.0001	235 (4.6%)	206 (3.9%)	+0.7% (−0.1, 1.4)	0.0993
Mortality, n (%)	8709 (7.3%)	8102 (6.8%)	+0.5% (0.3, 0.7)	<0.0001	6733 (12.5%)	6635 (12.3%)	+0.2% (−0.2, 0.6)	0.2645	690 (12.3%)	760 (13.5%)	−1.2% (−2.4, 0.1)	0.0681

Cohort 1: BP > 160 mmHg); Cohort 2 (140–159 mmHg), RD: Risk Difference % (95% CI).

## Data Availability

Data are extracted from TriNetX database available at https://trinetx.com/.
